# Low Dose Aerosol Fitness at the Innate Phase of Murine Infection Better Predicts Virulence amongst Clinical Strains of *Mycobacterium tuberculosis*

**DOI:** 10.1371/journal.pone.0029010

**Published:** 2012-01-03

**Authors:** Neus Caceres, Isaac Llopis, Elena Marzo, Clara Prats, Cristina Vilaplana, Dario Garcia de Viedma, Sofía Samper, Daniel Lopez, Pere-Joan Cardona

**Affiliations:** 1 Unitat de Tuberculosi Experimental, Fundació Institut per a la Investigació en Ciències de la Salut Germans Trias i Pujol, Universitat Autònoma de Barcelona, Badalona, Catalonia, Spain; 2 CIBER Enfermedades Respiratorias, Bunyola, Balears, Spain; 3 Departament de Física i Enginyeria Nuclear, Escola Superior d'Agricultura de Barcelona, Universitat Politècnica de Catalunya, Castelldefels, Catalonia, Spain; 4 Servicio de Microbiología Clínica y Enfermedades Infecciosas, Hospital Gregorio Marañón, Madrid, Spain; 5 Instituto Aragonés de Ciencias de la Salud, Zaragoza, Spain; 6 Hospital Universitario Miguel Servet, Zaragoza, Spain; Charité, Campus Benjamin Franklin, Germany

## Abstract

**Background:**

Evaluation of a quick and easy model to determine the intrinsic ability of clinical strains to generate active TB has been set by assuming that this is linked to the fitness of *Mycobacterium tuberculosis* strain at the innate phase of the infection. Thus, the higher the bacillary load, the greater the possibility of inducting liquefaction, and thus active TB, once the adaptive response is set.

**Methodology/Principal Findings:**

The virulence of seven clinical *Mycobacterium tuberculosis* strains isolated in Spain was tested by determining the bacillary concentration in the spleen and lung of mice at weeks 0, 1 and 2 after intravenous (IV) inoculation of 10^4^ CFU, and by determining the growth *in vitro* until the stationary phase had been reached. Cord distribution automated analysis showed two clear patterns related to the high and low fitness in the lung after IV infection. This pattern was not seen in the *in vitro* fitness tests, which clearly favored the reference strain (H37Rv). Subsequent determination using a more physiological low-dose aerosol (AER) inoculation with 10^2^ CFU showed a third pattern in which the three best values coincided with the highest dissemination capacity according to epidemiological data.

**Conclusions/Significance:**

The fitness obtained after low dose aerosol administration in the presence of the innate immune response is the most predictive factor for determining the virulence of clinical strains. This gives support to a mechanism of the induction of active TB derived from the dynamic hypothesis of latent tuberculosis infection.

## Introduction

The concept of virulence has been interpreted from both a host and pathogen point of view [1]. As far as *M.tuberculosis* is concerned, it was predominantly defined and determined in terms of survival and histopathology from the beginning of the 20th century [2] until the 1990s, when virulence begun to be measured by determining the growth rate [3] according to the Darwinian concept of virulence: “likelihood to survive and reproduce” [4]. Virulence in *M.tuberculosis* is therefore commonly determined by its maximum growth rate, which is expressed numerically by the slope of the regression curve in a semi-logarithmic representation of bacterial growth and is consensually called “Fitness”.

Although the virulence of strains involved in human outbreaks could originally only be deducted from epidemiological retrospective studies, experimental models are now used. The first studies regarding the virulence of *M.tuberculosis* were performed using *in vivo* models [5]. However, although animal models are still used to determine virulence [6], new approaches using tissue cultures (macrophages, dendritic cells and pneumocytes) [7] and *in vitro* liquid cultures using the Bactec and the MGIT systems [8], [9], have been designed. Virulence studies are highly focused on analyzingthe increase or decrease of the fitness of drug-resistant clinical and mutant strains. These emerging drug-resistant strains are studied retrospectively from an epidemiological point of view and their virulence is subsequently characterized experimentally. These studies have shown that there is a strong selection pressure for drug-resistance-conferring mutations that cause minimal fitness defects [4]. Moreover, W-Beijing strains have shown to be specially virulent in animal models of infection [11]. Indeed, certain lineages have been related to an increased transmissibility and/or pathogenicity [12], [13], and it has been suggested that there is a correlation between the virulence parameters and epidemiological characteristics of new Beijing strains [14].

Previous literature reports encouraged us to develop a new quick and easy experimental model to test the virulence of clinical strains in order to predict the potential damage that these strains could cause in a population. As results from our previous experiments suggested that a constant endogenous reinfection occurs during Latent Tuberculosis Infection (LTBI) [15], we considered that the speed of growth before the onset of the specific immune response, together with reinfection in the upper lobe, where a higher oxygen pressure promotes faster bacillary growth, could be crucial for the infection to progress to active disease. In this context, the higher the bacillary load the higher the probability that it will induce liquefaction (and posterior cavitation) once the immune response appears [16] [17]. Because of this original approach to the natural history of tuberculosis (TB), we also consider the maximum growth rate between 0 and 2 weeks post-inoculation to be the best means of determining the virulence of a strain.

A simple and short time-framed murine model was developed and validated by testing *M. tuberculosis* clinical strains with demonstrated epidemiological importance in Spain in the last 20 years in three Spanish regions (Grand Canaria, Madrid and Zaragoza). The development of this virulence model led to a need to determine the quality of the inoculum by studying the characteristics of its cording properties, i.e., the *M. tuberculosis* tendency to join into bacillary aggregates with a parallel orientation of cells' axes [18], [19]. This determination resulted in a valuable “*in vitro*” test to predict the “*in vivo*” behavior. Two different virulence rankings were obtained in the *in vivo* models depending on the infection route used. The results obtained suggest this is because the low-dose aerosol model (AER) mainly describes the fitness of isolated bacilli, whereas the intravenous model (IV) gives information regarding both isolated and corded bacilli. The AER model has been found to best fit the dissemination of TB cases in the community. This fact reinforces the concept that a higher fitness at the beginning of the infection process correlates with the ability to induce TB disease, thus supporting the concept derived from the dynamic hypothesis of LTBI.

## Results

### *In vitro* fitness clearly favors the standard laboratory strain

All strains grew in 7H9 Middlebrook supplemented medium as expected except for strain 7, which, although it was cultured three times, showed no evidence of growth during the period of time in which it was previously reported to do so (i.e. 1 month). For this reason, no CFU data from the Strain 7 were used in the fitness calculation.

**Figure 1:**
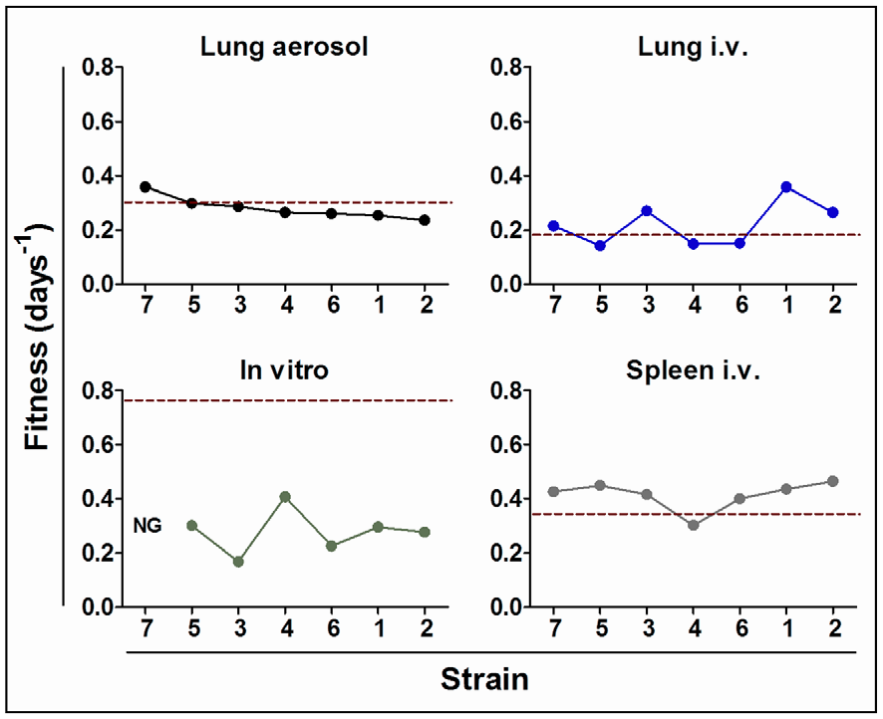
Fitness results. Fitness results from *in vivo* and *in vitro* studies. Top: fitness values for lung aerosol and IV experiments; bottom: *in vitro* assay and spleen IV infection values. Dotted lines indicate the fitness value for the H37Rv strain (shown as “St”).

The fitness of the reference strain (H37Rv Pasteur) was considerably higher than that for the other strains (Figure 1), followed, in decreasing order, by the clinical strains (Table 1). Remarkably, isolates 4 and 5 (actually the same strain) showed a similar fitness, thus giving some robustness to this method. No correlation was found between fitness and lag phase (data not shown).

**Table 1:** Fitness ranking in the different models.

	Fastest (left) to Slowest (right)							
***In vitro***	H37Rv	4	5	1	2	6	3	
**IV spleen**	2	5	1	7	3	6	H37Rv	4
**IV lung**	1	3	2	7	H37Rv	6	4	5
**AER lung**	7	H37Rv	5	3	4	6	1	2

Numbers correspond to strains detailed in Table 3. Strains are arranged from fastest (left) to slowest (right) in a decreasing order according to Fitness.

### The fitness in lungs after intravenous (IV) inoculation appears to be a reliable virulence method

**Figure 2:**
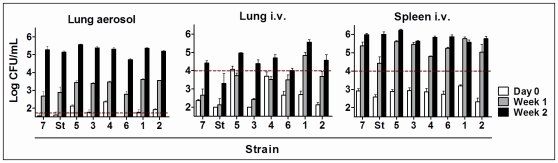
Fitness in the *in vivo* models. Bacillary load in the lung from aerosol-infected mice and lung and spleen from IV-infected mice on day 0, week 1 and week 2. The H37Rv strain is indicated as St. Dotted lines show the inocula used to infect each mouse.

The first *in vivo* model in mice involved infecting the animals by the intravenous route (IV). The bacillary load was determined in lung and spleen at day 0 and weeks 1 and 2 post-infection (Figure 2).

Spleen fitness results were higher than the corresponding values for lung fitness in all cases (Figure 1), whereas the lag phase was 0 in all strains (data not shown). Interestingly, all strains except strain 4 showed a higher fitness than H37Rv in the lung (Table 1). Strains 4 and 5 showed totally different fitness, thus suggesting a poor robustness for this method.

No relation could be established between lung fitness and lag phase (data not shown). The H37Rv strain showed a medium position in the fitness ranking (Table 1), and isolates 4 and 5 showed a similar fitness, which was the lowest.

### The cording phenomenon is related to fitness in the IV model

**Figure 3:**
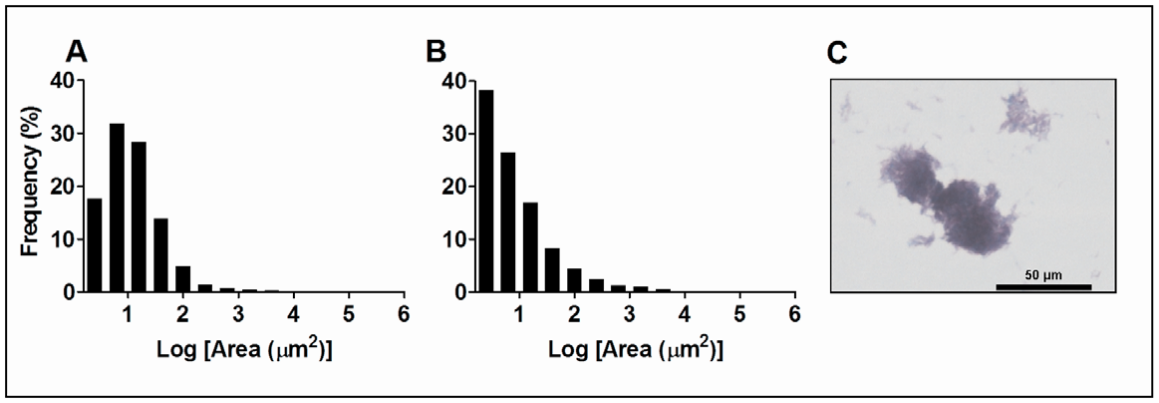
Aggregate area distribution results. Cording area analysis results for the strains inoculated, as obtained by image analysis. A and B: representation of aggregate area distribution showing the two characteristic patterns found. A: log-normal distribution observed for strain 3 (included in the fast strains group). B: decreasing exponential distribution observed for strain 5 (included in the slow strains group). C: picture from a Ziehl-Neelsen stained strain 1 sample showing different sized cords. Original magnification: 200×.

Cording was detected in all strains from the frozen late-log phase stocks of each strain used to inoculate each experimental model. Areas from single and aggregated bacilli in large cords from the frozen stocks of each clinical strain were analyzed by image analysis. The graphical representation of the area distribution of all strains displayed an asymmetric shape (Figure 3A and B). Two types of area distribution patterns were distinguished: the first pattern displayed a log-normal distribution whereby the maximum number of aggregates (any particle detected, from single bacilli to large cords) corresponds to the second column of the graph, which comprises the area between 2.5 and 6.3 µm^2^ (few bacillary aggregates, up to 5 bacilli). An exponential decrease is observed from 6.3 µm^2^ to higher areas (Figure 3A). The second pattern displayed a monotonous exponential decrease in which the maximum number of aggregates corresponds to the first column presented in the graph, which comprises the area between 1 and 2.5 µm^2^(single bacilli). An exponential decrease is observed from 2.5 µm^2^to higher areas (Fig. 3B). It is important to compare these data with the fact that the size of infective aerosols is around 7 µm^2^ [20].

**Figure 4:**
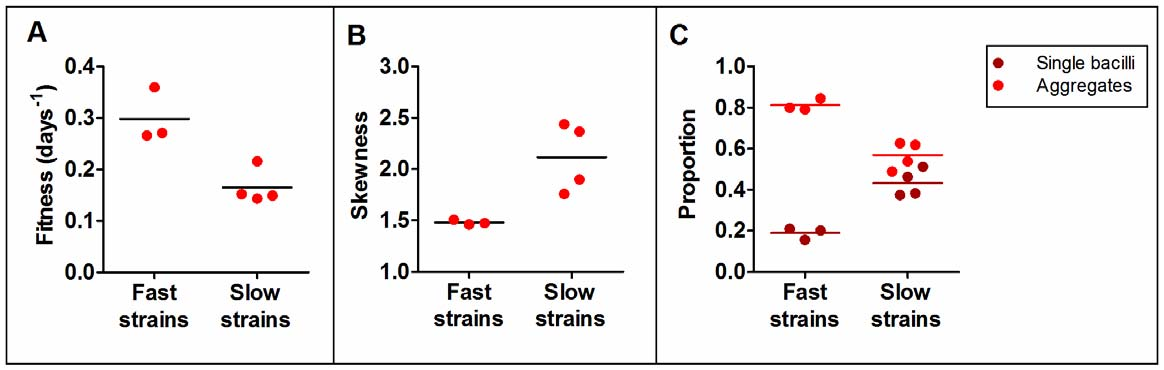
Division of clinical strains into fast and slow strains. The strains were divided in fast (1, 2 and 3) and slow strains (4, 5, 6 and 7) according to their fitness in lung in the *in vivo* model IV (panel A). Panel B and C show the representation of fast and slow strains according to parameters determined from the cording area analysis: skewness (panel B), and proportion of single bacilli (brown circles) and of aggregates (bright red circles) (panel C). A significant difference was found between fast and slow strains for all three graphs.

Once the clinical strains had been classified into the first or second pattern, statistical analysis of these two groups showed significant differences between them for the parameters lung fitness from IV infection (Figure 4A), skewness (a statistical parameter that indicates the asymmetry of a probability distribution; Figure 4B), cording proportion and single bacilli proportion (Figure 4C). As far as the lung fitness results for the IV infected mice are concerned, strains were grouped as fast (highest fitness values: strains 1, 2 and 3) or slow (lowest fitness values: strains 4, 5, 6 and 7). The H37Rv standard was also classified as a fast strain. The fast strains showed a clearly higher cording proportion and lower single bacilli proportion than slow strains.

### The AER model gives a third fitness ranking that is related to the epidemiologic data rather than the cording ability

Summarizing the previous data, the fitness obtained in lung after IV infection showed the best profile in terms of determining the virulence in clinical strains. In light of this, the information provided by the cording distribution, which correlates with the highest or lowest virulence, represents a notable achievement as this *in vitro* model could be a useful, quick and reliable matter for determining this parameter in large numbers of samples. In this regard, we planned to validate these results by testing the most physiological method, namely low-dose aerosol infection (AER), to better mimic the human infection in the lung (Figure 2).

The data obtained using the AER model gave a new fitness ranking (Table 1), with all values being within the “high virulence” range obtained using the lung IV model (Figure 1). In this model, the H37Rv standard showed the second highest value. Interestingly, the fitness in lung showed a significant correlation with lag phase, and isolates 4 and 5 showed similar results in terms of fitness (within the error margins for this technique; Table 2).

**Table 2:** Comparison of the fitness parameters according their duplication time in hours.

Model	Max	Min	Mean	SD	% CV
IV Spleen	23.88	15.54	18.27	2.68	14.70
*in vitro*	42.78	9.55	24.60	9.85	40.06
Aer. Lung	30.50	20.14	25.87	3.21	12.41
IV Lung	50.49	20.12	35.92	11.27	31.38

Table 2 shows a comparison of the fitness parameters for the strains according to their duplication time in all the experimental models used. This comparison shows how the lowest doubling time (best fitness) was obtained in the spleen after IV Inoculation, followed by *in vitro*, lungs after AER and lungs after IV, with spleen growth after IV inoculation and lung growth after AER being the models in which the lowest inter-strain variation was obtained. The *in vitro* model showed the highest variance mostly because of the low doubling time detected for the standard laboratory strain H37Rv (9.55 h) and the huge amount of time required by M3 strain (42.78 h), us considering these variances being due to the adaptation of each strain to the in vitro growth.

**Figure 5:**
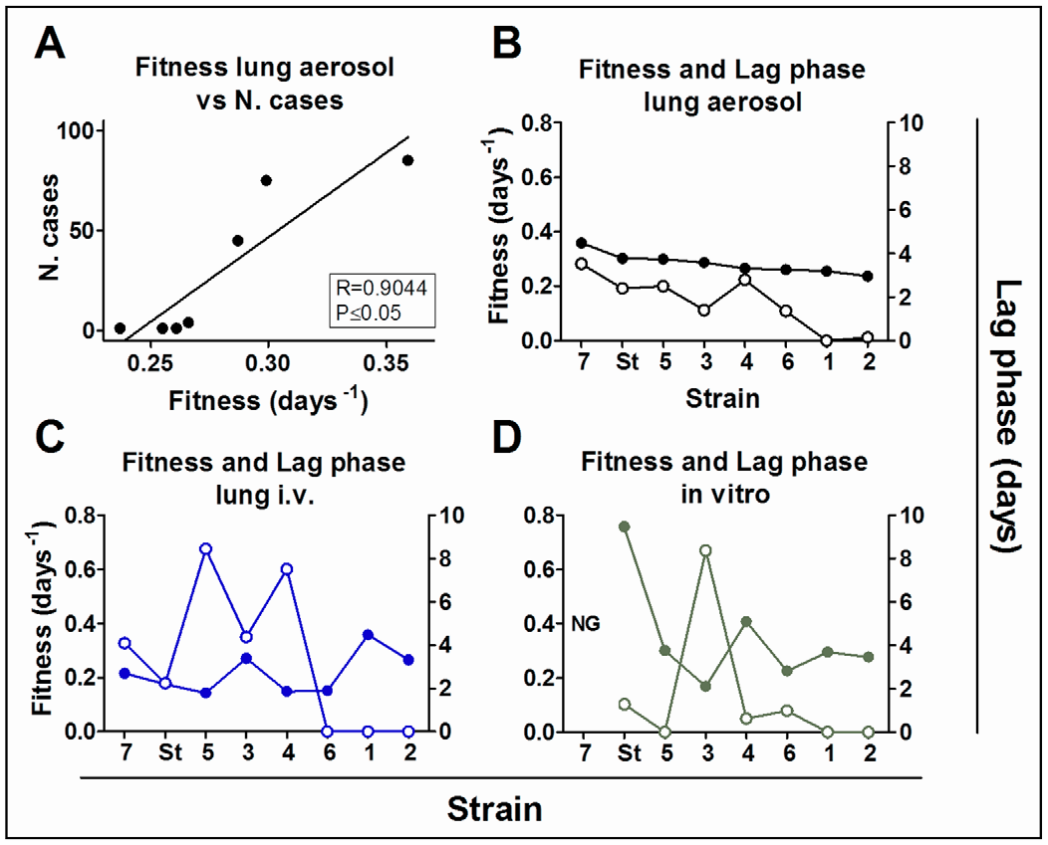
Fitness and Lag phase results. Comparison of fitness values from the different experiments with respect to the number of clinical cases detected (A) and the evaluated lag phase(B–D). A: correlation between fitness values from lung aerosol infections and the number of clinical cases reported, which resulted significant; B–D: fitness (left axes, filled circles) and lag phase (right axes, unfilled circles) of each experiment with respect to the different strains in the lung after aerosol infection (B), lung after IV infection (C) and *in vitro* assay (D).

The AER model was the only one to give a good correlation with the epidemiological data. A correlation was found between the calculated fitness in the *in vivo* AER model and the number of TB cases related to each strain (Figure 5). Thus, the AER model gave the maximum value to strain 7, which appears to be the strain with the highest capacity to disseminate throughout the population with the fewest associated risk factors (Tables 3 and 4).

**Table 3:** Features of the infectious agent.

Identification	Strain n	Beijing Lineage	Location of diagnosis	Period	Mϕ virulence level	n secondary cases	Ab resistant
Madrid 1	1	YES	Madrid		High	-	NO
Madrid 2	2	YES	Madrid		High	-	INH-R
Madrid 3	3	NO	Madrid	1997–2004	Low	45	NO
Madrid 4	4	YES	Madrid*		Low	-	NO
GC 1237	5	YES	Gran Canaria	1993–1996	ND	75	NO
HMS 1292	6	NO	Zaragoza		ND	-	NO
MTZ	7	NO	Zaragoza	2001–2004	ND	85	NO

Abbreviations: n: number; ND: Not Done; Mϕ: Macrophage; Ab: Antibiotic.

(*): Isolates 4 and 5 are representative of the same strain and corresponded to initial (Isolate 5, Gran Canaria) or final (Isolate 4, Madrid) stages of its dissemination.

**Table 4:** Epidemiological features of the host.

Identification	Male	Female	Age (years)	Immigrants*	Indigency	Previous TBC	Prison background	PDA	HIV	Transplant	Other
Madrid 1	0	1	2	Ecuador	-	NO	-	-	-	-	-
Madrid 2	1	0	51	Perú	-	YES (childhood)	-	-	-	-	DM II
Madrid 3	82.2%	17.8%	-	0,04	4.4%	-	26.7%	22.2%	0,4	2.2%	-
Madrid 4	-	-	-	-	-	NO	-	-	-	-	-
GC1237	-	-	35±14.8 (3 to 81)	0,19	0,12	-	0,16	0,39	0,35	-	-
HMS1292	1	0	over 18	YES	-	-	-	-	YES	-	-
MTZ	70.6%	29.4%	30^a^	0,14	-	-	0,05	0,07	15.3%	-	-

*: Country of origin.

a : (23 to 41 with percentil25–75) (8 months to 68 years).

Abbreviations: TBC: Tuberculosis; PDA: Parenteral drug abusers; HIV: Human immunodeficieny virus; DM II: Diabetes Mellitus II.

### None of the *in vivo* models induced a specific immune response

In order to ensure that the CFU values obtained in the mice experiments were due only to the growth capacity of each strain and were not influenced by the adaptive immune response of the host, a new experiment was performed, in which mice were infected IV with the reference or strain 1 (corresponding to high and low fitness values, respectively).

Neither ESAT-6 nor PPD-specific IFN-γ release could be detected by ELISPOT assay at any time point (day 0, week 1, week 2) in either spleen or hilar node samples of animals infected with any of the strains evaluated (Figure S1).

## Discussion

Conceptually, the virulence should be assessed considering the host and the pathogen, and it is true that at human level, every case should be thoroughly studied to exactly determine how much weight the host circumstances have in the evolution of the infection and the disease. However, at the experimental basis, we decided to focus in the pathogen behavior in the mouse model (thus in a precise host with concrete circumstances) taking into account the infection route (aerosol vs. intravenous), thus considering the concept of virulence determined by the growth rate and defined in the Introduction section.

This work aimed to establish an easy, quick and useful experimental model for analyzing the virulence of a recently isolated clinical *M. tuberculosis* strain. To validate this model, seven clinical strains isolated in Spain and of some epidemiological interest were evaluated together with a standard reference strain (H37Rv Pasteur).

All strains infected the host successfully, even if evident differences were encountered between the bacillary load found in the tissues and the inocula used to infect the animals, reflecting the different ability to successfully infect the host of the different strains. In general, the results obtained show that the fitness ranking is specific to each model. Thus, the fitness pattern provided by the *in vitro* model clearly favors the virulent standard strain H37Rv, which could be due to the fact that this strain was maintained subcultured for a long time until freeze-dry technology appeared in the 1960s. The reference strain showed a markedly higher value in the *in vitro* assay than in the *in vivo* studies. This might be due to antagonistic pleiotropy, which explains the fitness costs of adapting to a new system [21]. A rapid increase of parasite-induced reduction of host fitness is the most general result of serial passage experiments [22], and one passage through mice or guinea pig is not sufficient to restore the virulence [23]. The fitness values obtained *in vivo* and *in vitro* for the reference strain used herein fit with these statements, as H37Rv Pasteur is frequently cultured *in vitro* and is probably better adapted to grow in nutrient broth than clinical strains. For this reason, the use of the reference strain in experimental *in vivo* modeling has been also criticized [24]. The inability of strain 7 to grow in the 7H9 Middlebrook culture, and the fact that it achieved the highest position in the fitness ranking for the lung of AER-infected mice, reinforces the idea of this strain being probably more used to surviving and growing through human host passages rather than through culture medium. These results clearly show that the *in vitro* fitness is not a good model for predicting the virulence of *M. tuberculosis* strains.

As far as the *in vivo* models are concerned, it is important to note that the duplication time in lung after IV inoculation was the highest registered. As the lung appears to filter the larger particles after IV inoculation, this suggests that the bacilli inside cords require a longer time to growth. Likewise, these cords seem not to reach the spleen, which therefore mainly receives single bacilli, as is the case in lung after AER infection. The paradox is that when comparing the pulmonary growth of the different strains in the IV model, cording appears to favor a higher fitness. This means that other factors, such as the nature of the macrophages that phagocytose bacilli, which are less aggressive in the spleen than in the lung, may play a role [25].

The lag phase can be considered to be “the interval that elapses between the time of seeding and the time at which maximum rate of growth begins” [26] or as “the interval that elapses between the time of seeding and the time of detectable growth” [9], [10]. Determination of the lag phase contains an inherent error resulting from the different measurements used in experimental procedures, therefore we estimated this parameter by adapting the methodology of Prats et al. [27], [28], which estimates the lag phase on the basis of experimental data.

The lag phase has been used as an indicator of *M.tuberculosis* virulence only occasionally. Indeed, it is more often used to determine the fitness of *E.coli* [29], [30]. No correlation was found between the fitness and lag phase parameters for IV-infected mice and the *in vitro* assay in the present study. This lack of correlation could be due to the less frequent monitoring of CFU load in our experiments in comparison to the hourly monitoring of the BACTEC MGIT system in the Toungoussova paper, which found a trend in terms of growth rate in relation to lag phase [9]. In contrast, the lag phase results obtained with the lungs of AER-infected mice correlated with the corresponding fitness values. However, this correlation was positive, thus indicating that, in contrast to what we expected to find (high virulence has previously been associated with a short lag phase [9]), higher virulence results in a longer lag phase. This finding could be a result of the methodology used to calculate the lag phase, which does not consider the lag phase as the time at which the strain starts to grow but the time of intersection between the initial cell concentration and the prolongation of the exponential growth phase line in a semi-logarithmic representation of the growth curve [17], [27]. This might reflect the fact that, with no cording, a higher lag phase may be important to adapt to the stressful milieu of the macrophage.

The bacillary load in terms of CFU results obtained in the animal model suggested the AER experiment to be the best model for testing virulence as it showed a correlation with the fitness determined from reported lung and epidemiological data, expressed as number of diagnosed cases. It must be noted, however, that this fitness is obtained with no implication of the adaptive immune response. This concept comes from the dynamic hypothesis of latent tuberculosis infection, which proposes that a constant reinfection process is needed to maintain the infection and avoid the constant drainage of non-replicating bacilli. In this context, if this reinfection takes place in the upper lobe, where a higher growth is potentiated by the high oxygen pressure, this higher growth increases the likelihood of liquefaction (and thus active disease) once the adaptive response appears [16], [17].

The AER model is the only one that correlates with the physiological transmission of TB seen in humans, thus making it the gold standard of infection. This also fits with the idea that constant endogenous reinfection maintains the LTBI according to the dynamic hypothesis [16]. The differences found between this and the IV model might partially reflect the fact that the infecting aerosols measure less than 7 µm^2^, thus meaning that they harbor five bacilli each at most [20]. This model does not therefore consider the cord-forming ability of each strain, which must be taken into account in all experimental models in which the strain is inoculated directly, whether *in vivo* (intranasal, IV or intratracheal challenge) or *ex vivo* (macrophage infection). The cord-forming ability of each strain may play a role in all these cases.

Other authors have obtained a very good correlation between the survival time and the ability to disseminate active TB when using an intratracheal model to evaluate the survival time of mice after induction of massive pneumonic disease by directly inoculating 10^6^ CFU in the lung [14]. Likewise, interference from cording induction has been observed when evaluating the fitness of strains using the macrophage model, with the highest values being observed for strains 1 and 2, for example, which did not demonstrate any dissemination according to the epidemiological data. Interestingly, these strains were also classified as highly virulent on the basis of the cord formation and fitness obtained using our IV model, whereas they provided the lowest values in the AER model [31]. This fact suggests that cording ability appears not to be determinant in predicting virulence. Of the three more disseminated strains, #3 showed a high cording ability, whereas strains 7 and 5 had some of the lowest cording abilities. Interestingly, the laboratory strain H37Rv showed a totally unique phenotype, combining a high cording ability with a very good fitness in the AER model. Taking into account the ease of this *in vitro* technique, it would be interesting to evaluate the cording ability when trying to determine the fitness of clinical strains.

Cording ability was first described by Koch in 1882, who referred to these structures as “densely bunched and braided groups” [18]. Cording is characterized by the formation of tight bundles of bacilli in which the orientation of the long axis of each cell is parallel to the long axis of the cord [19]. Although Trehalose dimycolate (TDM) was first described as the only responsible for the cording phenomenon [32], it has been proven that alterations in cell wall (non related to mycolic acids) can lead to loss of cording property [33] [19].

Cord formation has been previously related to virulence [34], [35] as well as to pathogenicity [36], although non-pathogenic mycobacteria are also able to form cords [19].

Cording ability has also been related to biofilm formation, at least in *M.marinum* [37]. We follow the criteria established by Julian et al. [19], who considered cording to occur when bacillary aggregation follows a definite order, when deciding whether to use the term cord or aggregate to describe structures. When using shacked cultures under constant aeration in order to get homogeneous inoculums, the cording phenomenon evolves towards the formation of large coiled structures similar to cinnamon rolls, a clear consequence of the orbital mechanics that forces this self-coiling [unpublished results]. Biofilm formation is induced once spinning is stopped [data not shown].

Formation of a biofilm, which is the traditional means of obtaining BCG [38] in non-shacked cultures, has been related to the induction of tolerance or resistance against antibiotics [39], [40] as this is thought to be how bacilli persist in necrotic tissue [39]. Indeed we consider it necessary for the bacilli to grow under extracellular conditions. Currently unpublished findings from our lab [unpublished results] suggest that the culture of *M.tuberculosis* with a high dose of Tween 20 (×10) stops growth due to a delayed induction of cording. Moreover, the present study relates this particularity to a higher fitness once a high dose is inoculated in the lung. It could be considered that this high dose generates a strong inflammatory response, thus leading to a hostile environment for the bacilli. In contrast, the low dose AER is more discrete, and thus less prone to induce a global response. Cording would be the inducer and the defense against this hostile environment, thus making it a virulence property.

We should emphasize that extracellular growth also occurs in TB once liquefaction takes place during evolution of the granuloma towards the cavity [41]. In this context, the bacilli might also grow by forming cords, thus helping to generate more destruction in the lesion by increasing its size and evolving to a cavity.

Despite the correlation found between fitness in lungs from AER-infected mice and epidemiological data, it should be noted that the ability of the host to produce infected AER could influence the transmissibility degree of a clinical strain, as reinforced by studies performed in humans [42] and guinea pigs [43], [44]. On the other hand, a previous virulence study in rabbits suggested that the successful transmission of strain CDC1551 was due to its high transmissibility rather than its high virulence [45]. Moreover, Table 4 shows a significant proportion of cases associated with virulent strains 3 and 5 as being drug-users, HIV-infected and of prisoner background, reinforcing the weight of the socioeconomic factors. All this means that epidemiological dissemination data should not be based solely on the intrinsic virulence of the bacteria but also on the host's conditions as these govern the ability of the host to interact. This study offers new insights that should be taken into account when determining the virulence of *M.tuberculosis* strains. However, the *in vitro* fitness of *M.tuberculosis* strains should be interpreted cautiously due to the potential fitness costs of adapting to a new system. Though non conclusive, as further studies might be required to corroborate its predictable value, the AER infection mouse model presented herein has proved to be a reliable method for predicting the virulence of clinical strains, what should bring the opportunity to implement preventive measures on time. Finally, the cording phenomenon has been related to fitness for the first time as a result of an exhaustive analysis of the distribution pattern of bacilli into aggregates.

## Materials and Methods

### Mice

6–8-week-old female Balb/c specific-pathogen-free (*spf*) mice were obtained from Harlan Laboratories (Sant Feliu de Codines, Catalonia, Spain). The animals were shipped under suitable conditions, with the corresponding certificate of health and origin. Upon arrival, the mice were kept under controlled conditions in a P3 high security facility with sterile food and water ad libitum.

### Ethics

All animal procedures were approved and supervised by the Animal Care Committee of the Germans Trias i Pujol University Hospital and by the Department of Environment of the Catalan Government (approval number 4095). Mice were weighed and checked every week following aprotocol that monitored weight loss, apparent good health (bristled hair and wounded skin) and behaviour (signs of aggressiveness or isolation). Mice were euthanized with isoflurane.

### Bacteria

*M.tuberculosis* H37Rv Pasteur, Madrid1, Madrid2, Madrid3, Madrid4, GC1237, HMS1292 and MTZ strains were used in this study. They were isolated in Spain within the last 20 years and were selected by the Madrid and Zaragoza laboratories for inclusion in the study (Tables 3 and 4) on the basis of being highly prevalent (strain 3 [46], [47], [48]) their involvement in outbreaks (5 and 7 [46], [47], [48]), their being a repeated orphan strain through time (strain 6), or showing high virulence in the macrophage model (strains 1 and 2). Strains 4 and 5 were actually two clinical isolates of the same strain. The first caused a large outbreak in Gran Canaria (strain 5) and could still be isolated in Madrid some years later (strain 4).

H37Rv Pasteur was used as the reference strain in all studies. Both the names of the strains and the corresponding numbers assigned to them are shown in Table 2.

In order to standardize the pre-inoculation conditions, the frozen strains sent by the collaborating laboratories were intraperitoneally inoculated into Balb/c mice (3 mice/strain). The homogenized spleens from mice infected with each strain were cultured in Middlebrook 7H11 agar medium for 21 days. Visible colonies were then subcultured in Middlebrook 7H9 aerated and stirred broth kept at 37°C and supplemented with 0.2% glycerol, 0.5% albumin-dextrose catalase (Becton Dickinson) and 0.05% Tween 80. Bacteria were left to grow to a late-log phase and stored at −70°C in 3 mL aliquots.

### *In vitro* experiment (Control of bacillary growth *in vitro*)

Each strain was cultured once in a Middlebrook 7H9 broth (150 mL) supplemented with 0.2% glycerol, 0.5% albumin-dextrose catalase (Becton Dickinson) and 0.05% Tween 80 (final concentration of 10^5^ CFU/mL) and kept at 37°C with constant aeration and stirring. Samples were extracted three times a week in order to analyze the bacillary load.

### *In vivo* experiments

The infection solution was prepared by diluting the frozen aliquoted strains with sterile PBS to obtain a final concentration of 10^6^ CFU/mL for the AER and 5×10^4^ CFU/mL for the IV infection solution. In the AER experiment, the animals were infected by inoculating 20–50 bacilli into the lungs using a Middlebrook device. In the IV experiment, a total volume of 0.2 mL containing 10^4^ CFU was inoculated to each mouse IV. Both the *in vivo* experiments were conducted once, including six mice per time point and experimental group. The number of viable bacteria in lung and spleen homogenates was measured at day 0, week 1 and week 2 by plating duplicated serial dilutions of the tissue samples. Special care was taken not to include hilar lymph nodes during removal of the lung in order not to artificially increase the CFU value.

For the adaptive immune response characterization, six mice were inoculated IV with H37Rv Pasteur and strain 1. Animals were euthanized at day 0, week 1 and week 2, when 250,000 PBMCs from spleen and hilar node were stimulated for 18 hours with PPD (Statens Serum Institute, Copenhague) and ESAT-6 (Lionex Therapeutics & Diagnostics Ltd., Braunsweig, Germany) at a concentration of 20 and 10 µg/mL, respectively. An ELISPOT assay was performed to determine the specific-IFN-γ secreted by using a MABTECH murine IFN-γ ELISPOT kit (MABTECH, Sweeden) according to the manufacturer's recommendations. The Spot Forming Units (SFU) were subsequently counted using an ELISPOT reader.

### Bacillary load

The bacillary load of samples obtained in both the *in vitro* and *in vivo* experiments was determined by culturing the samples on Middlebrook 7H11 agar plates (Becton Dickinson, Madrid, Spain) at 37°C for 21 days. Visible colony forming units (CFU) were counted and the bacillary load expressed as CFU/mL in each organ.

### Virulence parameters

The fitness parameter was defined as the maximum growth rate in the semi-logarithmic representation of bacillary load (log CFU/mL) versus time (days). This maximum slope was determined by performing a linear regression between the two consecutive time points that framed the maximum growth. The same definition was used for both *in vitro* and *in vivo* fitness assessments.

The lag phase parameter of an *in vitro* culture is usually considered as the intersection between the initial cell concentration and the prolongation of the exponential growth phase line in a semi-logarithmic representation of the growth curve [27]. This intersection occurs when the horizontal line resulting from the initial CFU concentration (y = b_1_) and the line resulting from the maximum growth in exponential phase (y = ax+b_2_) share the same y value, which is the same CFU concentration; b_1_ = ax+b_2_ where *b_1_* is the CFU/mL at the initial time point, *a* the slope and *b_2_* the Y-intercept when x = 0 and *x* the lag phase. Then, lag phase = (b_1_−b_2_)/a.

Thus, the lag phase for both *in vitro* and *in vivo* growth was assessed as the intersection between the prolongation of the maximum slope line defined above and the initial level. When the fitness calculation occurred between time point 0 and the next, the Lag phase was considered to be 0.

### Image analysis of bacilli aggregates

A total of two 20 µL drops from the strains frozen at −70°C in the late-log growth phase (see 4.3.-Bacteria) were fixed on a glass slide and stained following the Ziehl-Neelsen procedure. A total of 8 to 10 photographs were taken using an Eclipse 50i microscope (Nikon, Japan) and a DS-Fi 1 camera (Nikon, Japan) at 100× using the Nis Elements imaging software (Nikon, Japan). Aggregates were then detected by image analysis using MATLAB software (MATLAB, vs 7.9.0.529; The MathWorks™). The original colored image was first converted into a gray scale image, then a threshold was fixed to convert the image into a black and white one, with isolated black regions being considered to be “aggregates” (these being any particle detected, from single bacilli to large cords). The area of each aggregate was determined and the following parameters calculated automatically for each strain:

1. Area distribution: frequency of aggregates in 16 area intervals, logarithmically distributed between 1 and10^6^ µm^2^. The first column in the area distribution graphics corresponds to the areas between 1 and 2.5 µm^2^, which we considered to represent single bacilli.
2. Skewness: a statistical parameter that measures the asymmetry of the frequency distribution and is positive when the tail on the right side is longer than on the left side and the bulk of the values lie to the left of the mean (and vice versa).
3. Cording proportion: ratio between the number of cords and the number of total aggregates, with a cord being defined as the aggregates in the second column or those with an area greater than 2.5 µm^2^.
4. Single bacilli proportion: ratio between the number of single bacilli and the number of total aggregates, with single bacilli being defined as the aggregates in the left-hand column or those smaller than 2.5 µm^2^. This parameter can also be defined as: (1 – cording proportion).
5. Mean area: average area of all the aggregates detected.
6. Maximum area: size of the largest area detected.

### Cording stain

Slides containing two 5 µL samples from exponential phase H37Rv cultures were stained using the BacLight™ Bacterial Viability Kit (Invitrogen, Carlsbad, CA) according to the manufacturer's instructions and observed using a Zeiss Axioskop epifluorescence microscope and the Axiovision Rel.4.8 software (Carl Zeiss, Madrid, Spain).

### Statistical analysis

Regression and Student's t analyses were performed using the GraphPad Prism Software (v4.03.354; La Jolla, California, USA). Differences were considered to be statistically significant at *p*<0.05.

## Supporting Information

Figure S1 **IFN-γ releasing PBMCs in the mice virulence study.** ELISPOT IFN-γ results from the spleen and hilar node of infected mice. The graphs on the left correspond to H37Rv-infected mice and those on the right correspond to mice infected with strain 1. Dotted lines indicate the threshold of negative IFN-γ assay, determined as the maximum value observed on day 0. No significant differences between day 0 and weeks 1 or 2 were detected.
(TIF)
