# Correction: Low Dose Aerosol Fitness at the Innate Phase of Murine Infection Better Predicts Virulence amongst Clinical Strains of *Mycobacterium tuberculosis*

**DOI:** 10.1371/journal.pone.0222794

**Published:** 2019-09-17

**Authors:** The PLOS ONE Staff

The sixth author’s initials appear incorrectly in the citation. The correct citation is: Caceres N, Llopis I, Marzo E, Prats C, Vilaplana C, García de Viedma D, et al. (2012) Low Dose Aerosol Fitness at the Innate Phase of Murine Infection Better Predicts Virulence amongst Clinical Strains of *Mycobacterium tuberculosis*. PLoS ONE 7(1): e29010. https://doi.org/10.1371/journal.pone.0029010
