# Supplementary figures and images for: Low Dose Aerosol Fitness at the Innate Phase of Murine Infection Better Predicts Virulence amongst Clinical Strains of Mycobacterium tuberculosis
Source: PLoS One. 2012 Jan 3;7(1):e29010. doi: 10.1371/journal.pone.0029010 (PMC3250398; doi:10.1371/journal.pone.0029010)

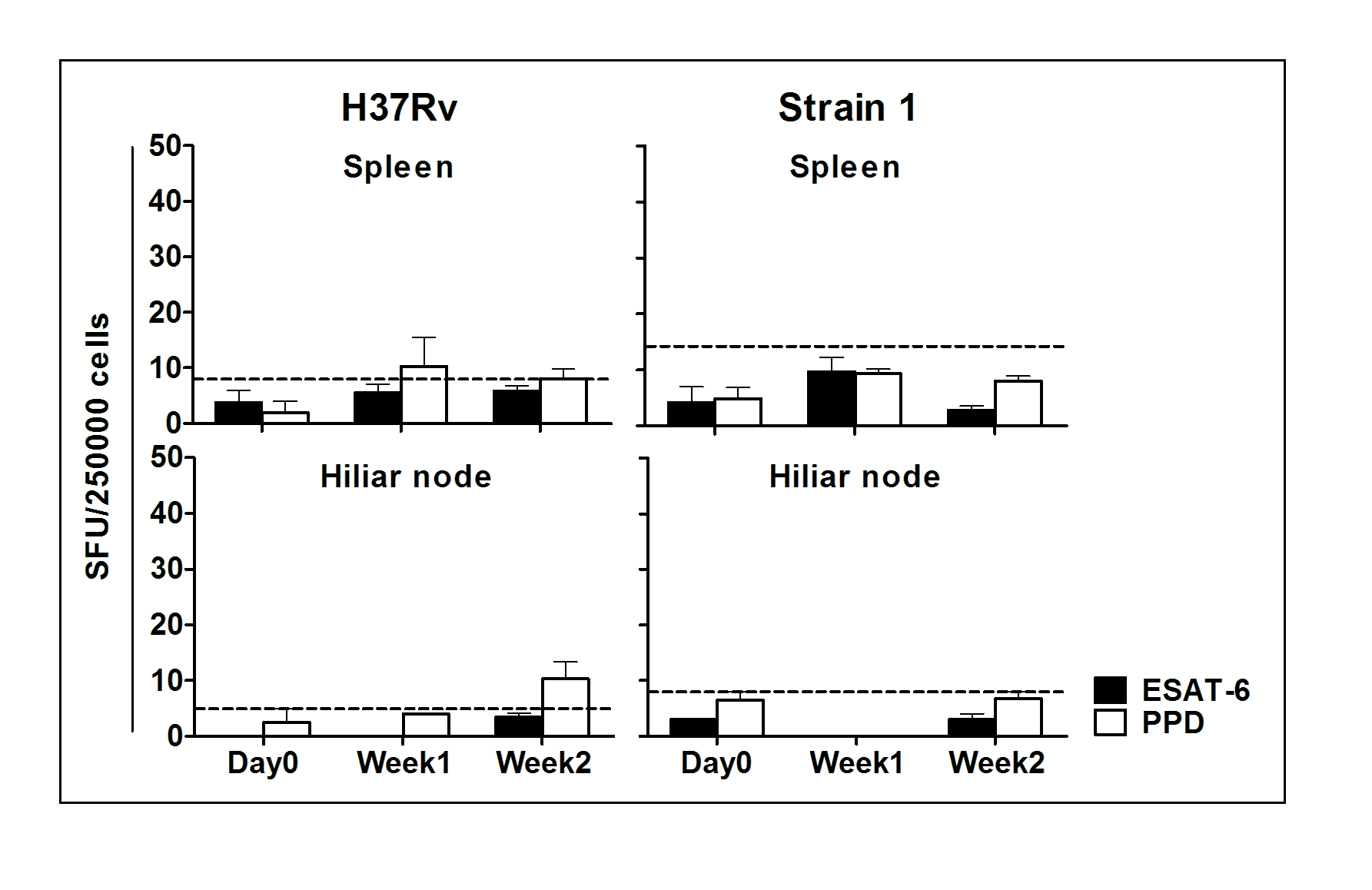

Supplement: Figure S1 — IFN-γ releasing PBMCs in the mice virulence study. ELISPOT IFN-γ results from the spleen and hilar node of infected mice. The graphs on the left correspond to H37Rv-infected mice and those on the right correspond to mice infected with strain 1. Dotted lines indicate the threshold of negative IFN-γ assay, determined as the maximum value observed on day 0. No significant differences between day 0 and weeks 1 or 2 were detected. (TIF) [file pone.0029010.s001.tif]
